# A Retrospective Chart Review Evaluating the Relationship between Cancer Diagnosis and Residential Water Source on the Lower Eastern Shore of Maryland, USA

**DOI:** 10.3390/ijerph18010145

**Published:** 2020-12-28

**Authors:** Angela DeRidder, Sowjanya Kalluri, Veera Holdai

**Affiliations:** 1 Department of Hematology and Oncology, TidalHealth Peninsula Regional, 100 E. Carroll St., Salisbury, MD 21801, USA; 2 Department of Internal Medicine, TidalHealth Peninsula Regional, 100 E. Carroll St., Salisbury, MD 21801, USA; sowjanyakalluri2@gmail.com; 3 Department of Math and Computer Science, Salisbury University, 1101 Camden Ave, Salisbury, MD 21801, USA; VXholdai@salisbury.edu

**Keywords:** drinking water, groundwater contamination, cancer risk, rural health, agricultural pollution

## Abstract

Well water contamination in heavily agricultural regions has previously been linked with increased cancer incidence and mortality. The lower Eastern shore of Maryland is a rural, agricultural region with some of the highest rates of cancer in Maryland and the United States. Our study sought to characterize residential private well water use among cancer patients on the lower Eastern shore of Maryland, and to compare private well water utilization between cancer patients and the general regional population. Retrospective chart review was conducted to identify patients diagnosed with colon, lung, melanoma or breast cancer at a regional hospital from 1 January 2017 through 31 December 2018. Residential water source was determined using residential address and municipal water records. Fisher’s exact test was used to compare residential private well water utilization between our study population and the baseline regional population. The majority of cancer patients (57%) lived in homes supplied by private well water (428/746). Cancer patients were more likely to live in homes supplied by private well water compared to individuals in the general regional population (57% vs. 32%, *p* < 0.001). In conclusion, cancer patients on the lower Eastern shore of Maryland were more likely to live in homes supplied by residential private well water than the regional population. Additional studies are needed to evaluate well water use and cancer risk in this vulnerable region.

## 1. Introduction

Per the Environmental Protection Agency (EPA), it is estimated that more than 13 million households rely on private wells for drinking water in the United States (U.S.) [1]. However, drinking water from private wells can pose potential health hazards. The EPA does not regulate private wells, and routine testing of private wells is voluntary and per the owner’s discretion [2]. Private well owners are therefore responsible for the safety of their own water supply.

As of 2018, the EPA recommends testing private wells annually for coliform bacteria, nitrates, total dissolved solids, and pH levels [2]. In addition, well owners should determine whether ground water supplying their private well is under direct influence from surface water. Substances such as nitrates/nitrites, arsenic, and organic chemicals can contaminate private wells through groundwater movement and surface water runoff [3]. Also known as non-point source pollution, surface water runoff is particularly concerning in heavily agricultural regions. Due to the common use of fertilizers and pesticides/herbicides, and the presence of animal waste and by-products, agricultural non-point source pollution is a leading cause of impaired water quality and ground water contamination in the U.S. [4,5].

Ground water contamination from agricultural sources can adversely affect human health in multiple ways. Elevated arsenic exposure in drinking water is associated with increased risk of skin, lung and bladder cancer [6,7]. Similarly, long term-exposure to nitrites and nitrates in drinking water has been associated with increased risk of colon cancer and breast cancer in certain patient populations [8,9,10]. Organic chemicals have been linked to a number of adverse effects on human health, including breast cancer [11]. While these contaminants can come from naturally occurring sources, such as arsenic deposits and wildlife waste products, studies have also shown that these potentially carcinogenic contaminants are found in a number of common agricultural byproducts and applications [12,13]. Rural, agricultural regions may therefore be at higher risk for groundwater contamination and its potential health implications.

**Figure 1:**
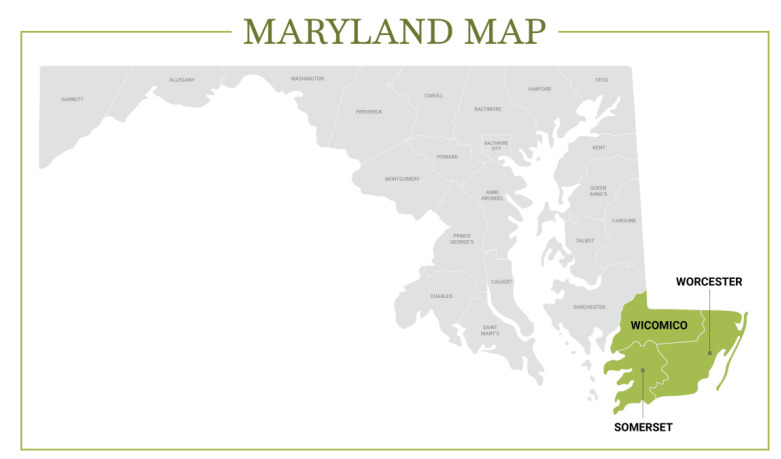
The lower Eastern shore of Maryland, U.S. Image created by MRamadhon and reproduced with permission.

The lower Eastern shore of Maryland lies on the east side of the Chesapeake Bay and consists of Wicomico, Worcester and Somerset counties (Figure 1). The main economic activities for this rural region include agriculture and large-scale chicken breeding. In 2014, Maryland ranked ninth among all states in the nation in broiler chicken production, with Wicomico, Worcester and Somerset counties being the top three producers of poultry and eggs within the state [14,15,16,17]. Approximately 600 million chickens are produced yearly between the lower Eastern shore of Maryland and the nearby county of Sussex, Delaware [18]. Per the EPA, these birds produce approximately 5.7 billion pounds of manure annually, contributing around 5 million pounds of nitrogen to local waterways per year. Combined with other agricultural sources on the lower Eastern shore, these farms, also known as animal feeding operations (AFOs), add a total of 119 million pounds of nitrogen pollution to the Chesapeake Bay on an annual basis. Given this documented contamination of local waterways by agricultural non-point source pollution, the economic activities prevalent on the lower Eastern shore of Maryland raise concern over the risk for ground water contamination in this region.

Local groundwater research suggests that these concerns may have merit. The U.S. Geological Survey (USGS) National Water-Quality Assessment Program measured concentrations of pesticides and herbicides in samples collected from 2001 to 2004 from 47 wells in Maryland, 16 of which were located on the Eastern shore [19]. Twenty-four out of 30 samples (80%) from the Eastern shore contained at least 1 detectable pesticide or degradate compound, and 16 (53%) contained at least 5 detectable compounds, with the majority of detected compounds being herbicides used for agriculture. One sample contained 11 different compounds. While no sample contained any pesticide or herbicide compounds at concentrations exceeding established Federal drinking water standards, such standards exist for only 4 out of all compounds detected (see supplemental data, Table S1 for list of detected compounds). More recently, researchers Minovi and Schmitt assessed private well water data from Wicomico and Worcester Counties to determine the extent of nitrate contamination on the lower Eastern shore. The researchers concluded that Wicomico and Worcester Counties have detected nitrates at levels exceeding the EPA’s safe drinking water threshold in approximately one out of every 25 private drinking water wells [20].

Cancer statistics for the lower Eastern shore of Maryland are also high. According to the U.S. Surveillance, Epidemiology, and End Results (SEER) database, Wicomico, Worcester, and Somerset counties have some of the highest rates of lung cancer, colorectal cancer, breast cancer, and melanoma in Maryland [21]. Wicomico and Somerset counties all sites cancer incidence rates from 2007 to 2011 were >25% above national rates, and all sites cancer incidence rates in Worcester county were 10–25% above national rates. In addition, the mortality rates for several types of cancers, including lung and colon cancer, are higher in Wicomico, Somerset and Worcester counties compared to national rates, suggesting more advanced disease at time of diagnosis.

Given the high incidence of several cancer types, the potential for ground water contamination, and the popularity of private well water use in this rural region, evaluating the relationship between cancer diagnosis and water source on the lower Eastern shore bears consideration. The purpose of this exploratory study was to characterize residential water source among patients on the lower Eastern shore of Maryland with a known diagnosis of melanoma, lung, breast, or colorectal cancer, and to determine characteristics, such as more advanced cancer stage, that might be associated with private well water use. We also compared residential private well water use among cancer patients in our study to private well water use for the general regional population.

## 2. Materials and Methods

### 2.1. Study Design

This study was approved through the Western Institutional Review Board. A retrospective chart review was conducted on all patients diagnosed with colon, lung, melanoma or breast cancer at a regional hospital from 1 January 2017 through 31 December 2018. This regional hospital serves as the largest hospital on the lower Eastern shore, with a catchment area that spans Wicomico, Worcester and Somerset Counties, as well as Sussex County in Delaware and Accomack County in Virginia. For most individuals living in these counties, the closest alternative hospital is located at least an hour away, and for some individuals, almost three hours away. While some individuals may seek subsequent or follow-up care outside of this regional hospital, the geographic isolation of the lower Eastern shore ensures that the majority of individuals living in this region seek initial diagnostic care locally. For example, there were 511 newly diagnosed cases of colon, lung, melanoma or breast cancer diagnosed in Wicomico, Worcester and Somerset Counties in the year 2016 [21]. According to institutional cancer registries, approximately 90% of these cases were diagnosed at this regional hospital [22].

Colon, lung, melanoma and breast cancers were selected for investigation because the incidence or mortality of each of these cancers is reported to be >10% higher on the lower Eastern shore compared to national rates [21]. Bladder cancer, a type of cancer that has been linked to agricultural pollution by other studies, was not included in our study due to low case numbers. Eligible patients were ≥18 years of age. Exclusion criteria included insufficient chart documentation, residential address outside of Wicomico, Worcester or Somerset counties, or atypical cancer diagnosis (angiosarcoma of the breast, colonic neuroendocrine tumor, etc.).

Age at time of cancer diagnosis, current residential address, sex, race and cancer type were recorded. Cancer stage, as well as smoking history, family history and prior cancer history were documented. Residential water supply was determined using the patient’s residential address and municipal water records.

### 2.2. Regional Private Well Water Statistics

Regional private well water utilization was determined using 2010 U.S. Census data, source water assessment documents for Wicomico, Worcester and Somerset Counties, as well as the comprehensive water and sewer plan for each county [23,24,25,26,27,28,29,30,31,32,33,34]. To determine the overall percentage of individuals on the lower Eastern shore who rely on residential private well water, we combined the number of individuals in each county relying on private well water and divided this by the total population for the region.

### 2.3. Data Analysis

Descriptive statistics were reported, including residential water supply for our total study population and for our study population stratified by county. Rates of residential private well water supply were compared between our study population and baseline regional population using the chi-square or Fisher’s exact test. All tests were two-tailed with a significance level of *p* < 0.05. Ability to control for confounding variables in our current exploratory study was limited due to the study design. Factors such as age, sex, and race were not controlled during our data analysis. We did evaluate the relationships between residential water supply and age of cancer diagnosis, cancer type, cancer stage, smoking history, family history and prior cancer history using Fisher’s exact test.

## 3. Results

### 3.1. Patient Characteristics

A total of 1248 charts were reviewed. A total of 420 cases had a residential address outside of Wicomico, Worcester and Somerset Counties (34%), and were therefore excluded from analysis. A total of 48 cases were excluded from analysis due to insufficient data (4%), 17 cases were excluded due to being duplicate cases (1%), and 17 cases were excluded due to rare cancer/non-cancerous diagnosis (1%). A total of 746 cases were ultimately eligible for analysis (60%). Baseline demographic characteristics and medical histories are summarized in Table 1. Sixty percent of patients resided in Wicomico County and 80% of patients self-identified as Caucasian. The median patient age was 67.2 years (SD ± 11.9). The majority of patients were female (67%), diagnosed with breast cancer (40%), had early stage cancer (63%), a prior or current smoking history (62%), a family history of cancer (68%), and no prior cancer history (68%).

### 3.2. Cancer Patient Private Well Water Utilization

The majority of cancer patients on the lower Eastern shore of Maryland lived in homes that relied on private well water (*n* = 428, 57%). At the county level, residential water source varied (Table 2). In Wicomico County, 64% of cancer patients lived in homes that relied on private well water (*n* = 285). In Worcester County, 41% of cancer patients lived in homes that relied on private well water (*n* = 81), while in Somerset County, 60% of cancer patients relied on residential private well water (*n* = 62).

### 3.3. Regional Private Well Water Utilization

According to the 2010 U.S. Census, 98,733 individuals live in Wicomico County [23]. Approximately 68% of housing units in Wicomico County, or 67,138 individuals, are on public or community water systems [24,25,26,27,28]. A total of 32 percent of housing units in Wicomico County, or 31,595 individuals are on private individual well systems.

According to communications from the Worcester County Department of Environmental Programs, anywhere between 67% and 80% of the 52,276 residents of Worcester County rely on public water systems [29,30,31,32,33]. For the purpose of this study, we estimated that an average of 73% (38,161) of Worcester County residents rely on public water systems, and 27% (14,115) rely on private well systems [23].

Somerset County is reported to have a population of 26,470 individuals [23]. The county estimates that approximately 5223 dwellings, or 12,706 individuals, receive drinking water from public municipal, county or community water systems (48%) [34,35]. Approximately 5555 dwellings, or 13,764 individuals, rely on private, individual water systems (52%).

With the three counties combined, we determined that 34% of individuals on the lower Eastern shore of Maryland rely on residential private well water (59,474/177,479).

### 3.4. Comparison of Private Well Water Use between Populations

**Figure 2:**
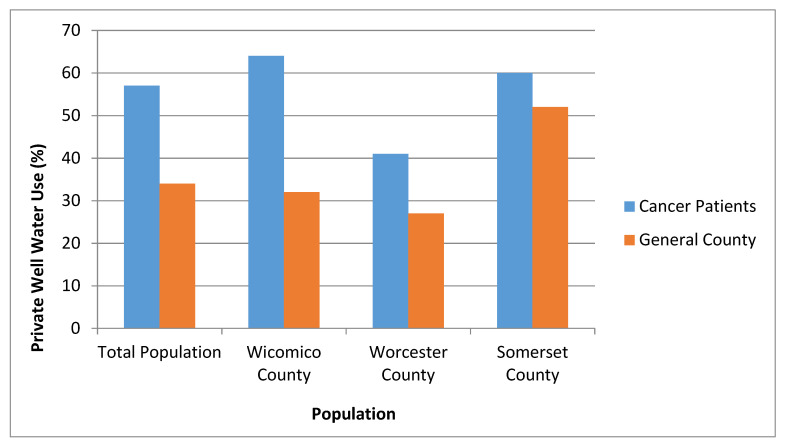
Residential private well water use for cancer patients compared to county population.

Our study found that on the lower Eastern shore of Maryland, cancer patients relied on residential private well water more than individuals in the general regional population (57% vs. 34%, *p* < 0.001) (Figure 2). Wicomico County cancer patients used residential private well water more than the general Wicomico County population (64% vs. 32%, *p* < 0.001). Similarly, Worcester County cancer patients used residential private well water more than the general Worcester County population (41% vs. 27%, *p* = 0.02). No significant difference was found between residential private well water use for Somerset cancer patients and the general Somerset County population.

### 3.5. Private Well Water Use and Patient Factors

Patients diagnosed with cancer at ≤70 years of age were more likely to rely on residential private well water compared to patients diagnosed over the age of 70 (61% vs. 52%, *p* = 0.01). There were no differences in sex, race, or family, smoking or prior cancer history between cancer patients using residential private well water versus public water (Table 3).

Cancer diagnosis type differed between patients relying on private well water versus public water, with higher frequencies of colon cancer and melanoma patients relying on private well water compared to patients diagnosed with breast and lung cancer.

Because secondary cancers can possibly be related to treatment toxicity from a previous cancer, a sensitivity analysis was performed to restrict analyses to only cases lacking a history of prior cancer diagnosis. Patient factors and residential water source were analyzed. Patients diagnosed with cancer at ≤70 years of age were again more likely to rely on residential private well water compared to patients diagnosed over the age of 70 (61% vs. 51%, *p* = 0.03). No new significant differences were noted in residential private well water use between our original analysis and our sensitivity analysis.

## 4. Discussion

### 4.1. Cancer Patients and Residential Private Well Water Utilization

Approximately 38% of the U.S. population depends on groundwater for its drinking water supply [36]. However, groundwater is susceptible to contamination. Due to the common use of fertilizers and pesticides/herbicides, and the presence of animal waste and by-products, agricultural non-point source pollution is a leading cause of impaired water quality and ground water contamination in the U.S. Previous studies have confirmed that in coastal watersheds, the amount of nitrate contamination found in groundwater is related to the proportion of agricultural land [36]. Rural, agricultural regions are therefore at higher risk for groundwater contamination and its potential health implications.

The lower Eastern shore of Maryland is a rural, heavily agricultural region that has a strong economic history of farming and large-scale chicken breeding. According to the EPA, over 600 million chickens are produced yearly on the Eastern shore and the neighboring county of Sussex, Delaware [18]. These birds, in turn, produce vast quantities of animal by-products including nitrates/nitrites, ammonia, arsenic and phosphorus. Contamination from these by-products has been shown to contribute to poor water quality of local surface waters, which in turn raises concerns over groundwater pollution. In fact, prior research has confirmed the presence of nitrates and agricultural pesticides in sampled well water from across the Eastern shore. While a full review of the detected groundwater contaminants on the Eastern shore is beyond the scope of this study, a brief list of contaminants previously detected by government agencies has been included in our supplemental data (see Tables S2 and S3 [19,24,25,26,27,28,30,31,32,33,34,35]).

Our study found that 57% of cancer patients on the lower Eastern shore use residential private well water, which is notably higher than the rate of private well water used nationally (14%), the rate of private well water used in the overall state of Maryland (19%), and the rate of private well water used by the lower Eastern shore population in general (34%) [1,36]. Though interpretation of our findings was limited by lack of causality, our study results were striking. Prior research has shown that proximity to farms and animal feeding operations is associated with increased risk for private well water contamination [37,38,39,40,41]. In addition, studies have shown that up to 50 percent of private wells tested do not meet at least one federal health-based drinking water standard [42,43,44,45,46]. Given the known risk of private well contamination in agricultural regions, as well as the known health implications of drinking contaminated well water, the association between private well water use and cancer diagnosis in this region warrants further investigation.

At the county level, our study found that patients living in Wicomico County and Worcester County relied on residential private well water more than general county statistics. Interestingly, Somerset County had no significant difference in residential private well water use between cancer patients and the general county population. This difference may stem from inter-county variation in policies regarding groundwater safety or animal feeding operations, and would need to be explored further in future studies.

Interestingly, our study found that cancer patients ≤70 years of age were more likely to rely on residential private well water compared to patients over the age of 70. These results should be interpreted with caution, as we did not control for potentially confounding variables. However, it is well established that advancing age is a risk factor for cancer development, while environmental exposure to carcinogenic materials can increase risk of cancer development at younger ages [47,48,49]. The association between well water exposure and younger age of cancer diagnosis in this region hints at a potentially carcinogenic relationship between these two variables, and again supports the need for additional studies examining the causality of private well water exposure and cancer risk in this region.

Finally, we found that cancer patients relied on residential private well water differently depending on their cancer diagnosis. All patients, regardless of cancer type, relied on residential private well water more than public water. However, colon cancer and melanoma patients used residential well water at higher frequencies compared to breast and lung cancer patients. Our ability to interpret these findings is limited though, given that we did not control for potentially confounding variables. Factors such as county of residence or age may account for these findings.

### 4.2. Limitations

Our study possessed several limitations. Due to the study design, we could not establish causality between residential private well water use and cancer diagnosis on the lower Eastern shore of Maryland. Importantly, our study did not sample and test the residential water supply of any of the patients included in this study. Without performing water testing, we cannot truly establish a connection between residential water source and cancer diagnosis. In addition, ability to control for confounding variables in our current exploratory study was limited due to the study design. Our study compared private well water use in cancer patients to general county statistics. While we were able to determine the percentage of each county that relied on residential private well water, we were unable to determine the age and race of this particular population at the county level, making controlling for these variables difficult. In addition, at the time of the study design, there was little historic data describing variables that might influence private well water use in this region. To preserve data accuracy, we did not wish to control for variables that may not be truly affecting our outcomes.

Diet was another factor that we were unable to evaluate and control for in our current study. It is well known that diet can influence cancer risk, and consuming carcinogenic foods, such as foods high in nitrates, can potentially increase cancer risk [50,51]. As this was a retrospective study, our ability to assess various patient factors was dependent on the data collected at the time of service. Extensive dietary review is not typically performed on patients diagnosed with cancer at this regional hospital. Similarly, extensive dietary information is not typically recorded at the county level. We were therefore unable to assess the extent dietary intake of potentially carcinogenic foods may be confounding our findings.

Because of the retrospective nature of this study, we were unable to confirm length of residency, or whether patients relied on residential tap water for their drinking water. In addition, while patient’s home address was determined via chart review, we were unable to confirm that the currently listed home address was also the patient’s home address at time of initial cancer diagnosis. Despite these limitations, however, this exploratory study brings to light an important public health issue, and provides grounds for further investigation.

### 4.3. Public Health Implications

To our knowledge, the current study is the first to evaluate private well water use on the lower Eastern shore of Maryland and its relationship with cancer diagnosis. At this time, the results of our study should not change local public or environmental policies, nor should they cause undue distress among local citizens who may consume private well water. While the results of our study are striking, they are not causal. As mentioned previously, we do not have data at this time confirming that private well water consumption in this region causes cancer.

That being said, the results of our study certainly signal a need for additional research on this topic. While our findings may simply be the result of confounding factors, alternatively they may truly reflect an increase in cancer risk for individuals on the lower Eastern shore who rely on residential private well water. A study published in 2018 by Schullehner et al. found an increased risk for colon cancer in persons exposed to higher levels of drinking water nitrate compared to persons exposed to lower levels [52]. Similarly, another study found that long term ingestion of elevated nitrate in drinking water was associated with increased risk of bladder cancer among postmenopausal women [53]. A meta-analysis performed by Saint-Jacques et al. in 2014 showed that arsenic in drinking water is associated with increased risk of bladder and kidney cancers [54]. These studies highlight the fact that drinking water research has the potential to impact the health of a large population, and its findings could have far reaching policy and monitoring implications.

### 4.4. Future Directions

The results of our study suggest that a relationship may exist between private well water use and cancer diagnosis on the lower Eastern shore of Maryland. Though there are many limitations to our study, our study certainly suggests that additional research is needed to clarify the relationship between these two variables. Additional steps to confirm this relationship would include a case-control study to compare well water use among cancer patients and individuals without a prior history of cancer. In this future study, determining if participants primarily drank tap water vs. bottled water, or used a drinking water filtration device, would be beneficial. In addition, determining how many glasses of tap water participants consumed per day, and the duration of home residency, would be interesting and provide further insight into this complicated yet highly relevant public health issue.

Alternatively, geographical variation in cancer incidence could be examined across the lower Eastern shore of Maryland. Spatial cluster analysis could be performed to determine whether spatial clusters in cancer incidence are associated with the presence of concentrated animal feeding operations or agricultural land use. This form of analysis can be difficult for diseases with complex etiology and long latency such as most cancers. Nonetheless, cancer cluster investigations in the past occasionally have led to the discovery of important pathways in the etiology of specific cancers, such as vaginal cell carcinoma and scrotal cancer [55,56].

Finally, additional research could include testing of private well water quality across the lower Eastern shore of Maryland for microbiological and chemical contaminants. One similar study, performed by Murray et al., examined private well water quality across four counties in Maryland. The study found that nearly half of tested wells did not meet federal health-based drinking water standards [42]. Interestingly, the study did not find a relationship between animal feeding operations and well water contamination; however, this study did not include well water samples from Wicomico, Worcester or Somerset counties.

## 5. Conclusions

In conclusion, the majority of cancer patients on the lower Eastern shore of Maryland relied on residential private well water, and cancer patients used residential private well water more than the general regional population. Younger cancer patients in this region were also more likely to rely on residential private well water compared to older cancer patients, though interpretation of these results should be made with caution due to inability to control for confounding variables. The findings of our study highlight the need for further research evaluating the relationship between well water exposure and cancer risk in this vulnerable region.

## Figures and Tables

**Table 1:** Demographics of Cancer Patients on the Lower Eastern Shore of Maryland; 1 January 2017–31 December 2018.

Characteristic		*n* = 745 (%)
Well Water Use		
	Yes	428 (57)
	No	317 (43)
County		
	Wicomico	444 (60)
	Worcester	198 (27)
	Somerset	103 (14)
Sex		
	Male	247 (33)
	Female	498 (67)
Age (years)		
	≤60	209 (28)
	61–70	241 (32)
	71–80	197 (27)
	≥81	86 (13)
Race		
	White	596 (80)
	Black	134 (18)
	Other	15 (2)
Diagnosis		
	Breast	297 (40)
	Lung	260 (35)
	Melanoma	106 (14)
	Colon	82 (11)
Stage		
	Early	471 (63)
	Advanced	273 (37)
Smoking History		
	Yes	462 (62)
	No	283 (38)
Family History of Cancer		
	Yes	517 (69)
	No	228 (31)
Prior History of Cancer		
	Yes	242 (32)
	No	503 (68)

**Table 2:** Residential Water Source for Cancer Patients on the Lower Eastern Shore by County; 1 January 2017–31 December 2018.

Characteristic	*n* = 745 (%)	
	Water Source	
	Private Well	Public Water
County		
Wicomico	286 (64)	159 (36)
Worcester	81 (41)	117 (59)
Somerset	62 (60)	41 (40)

**Table 3:** Patient Demographics for Cancer Patients on the Lower Eastern Shore by Residential Water Source; 1 January 2017–31 December 2018.

Characteristic		*n* = 745 (%)		*p*-Value
		Water Source		
		Private Well	Public	
Sex				
	Male	151 (61)	95 (39)	0.13
	Female	277 (56)	222 (44)	
Age (years)				
	≤70	276 (63)	174 (37)	0.01
	>70	152 (51)	143 (49)	
Race				
	White	346 (58)	250 (42)	0.69 **
	Black	75 (56)	59 (44)	
	Other	*	*	
Diagnosis				
	Breast	160 (54)	137 (46)	0.03 **
	Lung	143 (55)	117 (45)	
	Melanoma	69 (65)	37 (35)	
	Colon	56 (68)	26 (32)	
Stage				
	Early	271 (57)	201 (43)	1.0
	Advanced	157 (58)	116 (42)	
Smoking History				
	Yes	257 (56)	205 (44)	0.22
	No	171 (60)	112 (40)	
Family History of Cancer				
	Yes	298 (58)	219 (42)	0.87
	No	130 (57)	98 (43)	
Prior History of Cancer				
	Yes	136 (56)	106 (44)	0.64
	No	292 (58)	211 (42)	

* Data suppressed to preserve patient privacy. ** Differences between patients relying on private well water and public water were analyzed using Fisher’s exact test for all analyses except “Age” and “Diagnosis,” which were analyzed using the chi-square test.

## Data Availability

The data presented in this study are available on request from the corresponding author. The data are not publicly available in an effort to strictly ensure patient privacy.

## Supplementary Materials

The following are available online at https://www.mdpi.com/1660-4601/18/1/145/s1, Table S1: Pesticides and degradate compounds identified on the Eastern shore by the U.S. Geological Survey, 2001–2004. Adapted from data published by the U.S. Geological Survey [19], Table S2: Inorganic compounds, radionuclides, volitile organic compounds and synthetic organic compounds that were detected at least once either above 50% or 100% of their MCL in Wicomico County public water system (1991–2003) [24,25,26,27,28], Table S3: Inorganic compounds, radionuclides, volitile organic compounds and synthetic organic compounds that were detected at least once either above 50% or 100% of their MCL in Worcester 3County public water system (1991–2005) [30,31,32], Table S4: Inorganic compounds, radionuclides and volitile organic compounds that were detected at least once either above 50% or 100% of their MCL in Somerset County public water system (1994–2002) [34,35].
