# Supplementary material for: A Retrospective Chart Review Evaluating the Relationship between Cancer Diagnosis and Residential Water Source on the Lower Eastern Shore of Maryland, USA
Source: Int J Environ Res Public Health. 2020 Dec 28;18(1):145. doi: 10.3390/ijerph18010145 (PMC7796121; doi:10.3390/ijerph18010145)
Supplement: Supplementary file 1 [file ijerph-18-00145-s001.pdf]

International Journal of
Environmental Research and Public Health

MDPI

Supplemental material

Table S1. Pesticides and degradate compounds identified on the Eastern shore by the U.S. Geological Survey, 2001-2004. Adapted from data published by the U.S. Geological Survey [19].

**Pesticides**	**Degradate compounds**
Acetochlor ESA*	*2-Hydroxyatrazine**
Dinoseb*	*Alachlor ESA**
Flumetsulam*	*Alachlor OA**
Imazaquin*	*Deethyldeisopropylatrazine**
Imazethapyr*	*Deisopropylatrazine**
Imidacloprid*	*Dimethenamid ESA**
Propoxur*	*Flufenacet OA**
	*Metolachlor ESA**
	*Metolachlor OA**

*Substance has agricultural applications

Table S2. Inorganic compounds, radionuclides, volitile organic compounds and synthetic organic compounds that were detected at least once either above 50% or 100% of their MCL in Wicomico County public water system (1991-2003) [24-28].

**Substance**	**Detected at levels >50% MCL**	**Detected at levels >100% MCL**
IOC	Nitrate*	Nitrate*
IOC	Arsenic*	
IOC	Cadmium	
Radionuclide	Radon-222	Radon-222
VOC	Tetrachloroethylene	Tetrachloroethylene
VOC	Benzene	Benzene
SOC	1,2-Dibromo-3-Chloropropane	

MCL = Maximum contaminant level
IOC = Inorganic compound
VOC = Volatile organic compound
SOC = Synthetic organic compound
*Substance has agricultural sources/applications

Table S3. Inorganic compounds, radionuclides, volitile organic compounds and synthetic organic compounds that were detected at least once either above 50% or 100% of their MCL in Worcester 3County public water system (1991-2005) [30-32].

**Substance**	**Detected at levels >50% MCL**	**Detected at levels >100% MCL**
IOC	Nitrate*	
IOC	Arsenic*	
Radionuclide	Gross Alpha	
Radionuclide	Radon-222	
VOC	Benzene	Benzene
VOC	Methylene Chloride	
VOC	Methyl-tert-butyl-ether	Methyl-tert-butyl-ether
SOC	DI(Ethylhexyl)Phtalate	
SOC	Trihalomethanes	Trihalomethanes
SOC	Haloacetic Acids	

MCL= Maximum contaminant level
IOC = Inorganic compound
VOC = Volatile organic compound
SOC = Synthetic organic compound
*Substance has agricultural sources/applications

Table S4. Inorganic compounds, radionuclides and volitile organic compounds that were detected at least once either above 50% or 100% of their MCL in Somerset County public water system (1994-2002) [34-35].

Substance	Detected at levels >50% MCL	Detected at levels >100% MCL
IOC	Fluoride	Fluoride
IOC	Thallium	
IOC	Lead	
Radionuclide	Radon-222	

MCL = Maximum contaminant level [33,34].
IOC = Inorganic compound
VOC = Volatile organic compound
SOC = Synthetic organic compound
*Substance has agricultural sources/applications
